# Understanding Lithium intoxication in Bipolar Disorder: a comparative analysis and clinical implications

**DOI:** 10.1192/j.eurpsy.2024.622

**Published:** 2024-08-27

**Authors:** O. Martin-Santiago, C. D. Andres-Lobo, T. Jimenez-Aparicio, C. Vallecillo-Adame, A. Perez-Escudero

**Affiliations:** ^1^Hospital Clinico Universitario, Valladolid; ^2^Complejo Asistencial, Zamora, Spain

## Abstract

**Introduction:**

Lithium treatment is a proven method for bipolar disorder management, but its narrow therapeutic range and the risk of severe side effects, including lithium intoxication, pose significant clinical hurdles. Lithium intoxication, a potentially life-threatening complication, can occur during treatment, raising ongoing questions about its clinical factors, risk elements, and best practices for management.

**Objectives:**

Our objective is a comparative analysis between patients who have experienced lithium intoxication and those who have not, aiming to identify influencing factors and enhance clinical care.

**Methods:**

We collected demographic data, age at lithium treatment initiation, treatment duration, therapeutic adherence, Mental Health consultations, and lithium level monitoring from 14 individuals requiring clinical attention due to lithium intoxication and 14 patients with similar gender, age, and diagnosis with lithium treatment but without intoxication during four years of follow-up.

**Results:**

Regarding the results, the age of onset of lithium treatment in patients with lithium intoxication was 30.2 years (SD=8), and the duration of lithium treatment averaged 11.1 years (SD=8.8), which did not significantly differ from the control group with ages of onset at 38.1 years (SD=15.1) and treatment duration of 9.27 years (SD=8.8), respectively. Lithium intoxication patients developed severe complications, including hospitalizations in medical-surgical units, the necessity for dialysis, and death, one fatal case. Although therapeutic adherence to lithium, measured through pharmaceutical dispensation, exceeded 90% and was comparable in both groups, patients affected by lithium intoxication exhibited a significantly higher treatment discontinuation rate (OR 32.5; 95% CI, 3.1 to 337.8) during the follow-up period. Patients who experienced lithium intoxication had an average of psychiatric consultations every 11.2 months (SD=13.4), with 35.7% not attending at least once a year, while the control group had an appointment every 5.31 months (SD=2.7) (p > 0.05). Lastly, despite both groups having a similar frequency of plasma lithium level monitoring, occurring approximately every 5.5 months (SD=2.6) and 7.8 months (SD=4.8), respectively, in 28.5% of those who suffered from lithium intoxication did not undergo any monitoring for periods exceeding 18 months (p < 0.05).

**Conclusions:**

Our research highlights the significance of delivering thorough clinical care and continuous monitoring to patients receiving lithium treatment for bipolar disorder. Ensuring effectiveness therapeutic adherence and maintaining strict monitoring of lithium levels are critical factors that significantly enhance treatment safety. Appropriate management has the potential to improve the quality and safety of care for people with bipolar disorder who are dependent on lithium therapy.

**Disclosure of Interest:**

None Declared

